# Biophysical interactions between components of the tumor microenvironment promote metastasis

**DOI:** 10.1007/s12551-021-00811-y

**Published:** 2021-06-04

**Authors:** Dimitra Vasilaki, Athina Bakopoulou, Alexandros Tsouknidas, Elaine Johnstone, Konstantinos Michalakis

**Affiliations:** 1 Department of Prosthodontics, School of Dentistry, Faculty of Health Sciences, Aristotle University of Thessaloniki, University Campus, 54124 Thessaloniki, Greece; 2 Laboratory for Biomaterials and Computational Mechanics, Department of Mechanical Engineering, University of Western Macedonia, Kozani, Greece; 3 Department of Oncology, University of Oxford, Oxford, UK; 4 Division of Graduate Prosthodontics, Tufts University School of Dental Medicine, Boston, MA USA; 5 University of Oxford, Oxford, UK

**Keywords:** Metastasis, Circulating tumor cells, Cell mechanics, Cytoskeleton, Actomyosin contractility, Shear stress

## Abstract

During metastasis, tumor cells need to adapt to their dynamic microenvironment and modify their mechanical properties in response to both chemical and mechanical stimulation. Physical interactions occur between cancer cells and the surrounding matrix including cell movements and cell shape alterations through the process of mechanotransduction. The latter describes the translation of external mechanical cues into intracellular biochemical signaling. Reorganization of both the cytoskeleton and the extracellular matrix (ECM) plays a critical role in these spreading steps. Migrating tumor cells show increased motility in order to cross the tumor microenvironment, migrate through ECM and reach the bloodstream to the metastatic site. There are specific factors affecting these processes, as well as the survival of circulating tumor cells (CTC) in the blood flow until they finally invade the secondary tissue to form metastasis. This review aims to study the mechanisms of metastasis from a biomechanical perspective and investigate cell migration, with a focus on the alterations in the cytoskeleton through this journey and the effect of biologic fluids on metastasis. Understanding of the biophysical mechanisms that promote tumor metastasis may contribute successful therapeutic approaches in the fight against cancer.

## Introduction

Cancer continues to be a huge global health problem and one of the major barriers to human life expectancy. Data published by the World Health Organization (WHO) demonstrate that cancer is one of the two most frequent causes of death in more than half the countries of the world (World Health Organization 2018; Siegel et al. 2019). Mechanisms of cancer initiation, progression, and metastasis are still only partially understood; thus, research continues apace worldwide on the multifaceted nature of the disease.

**Fig. 1:**
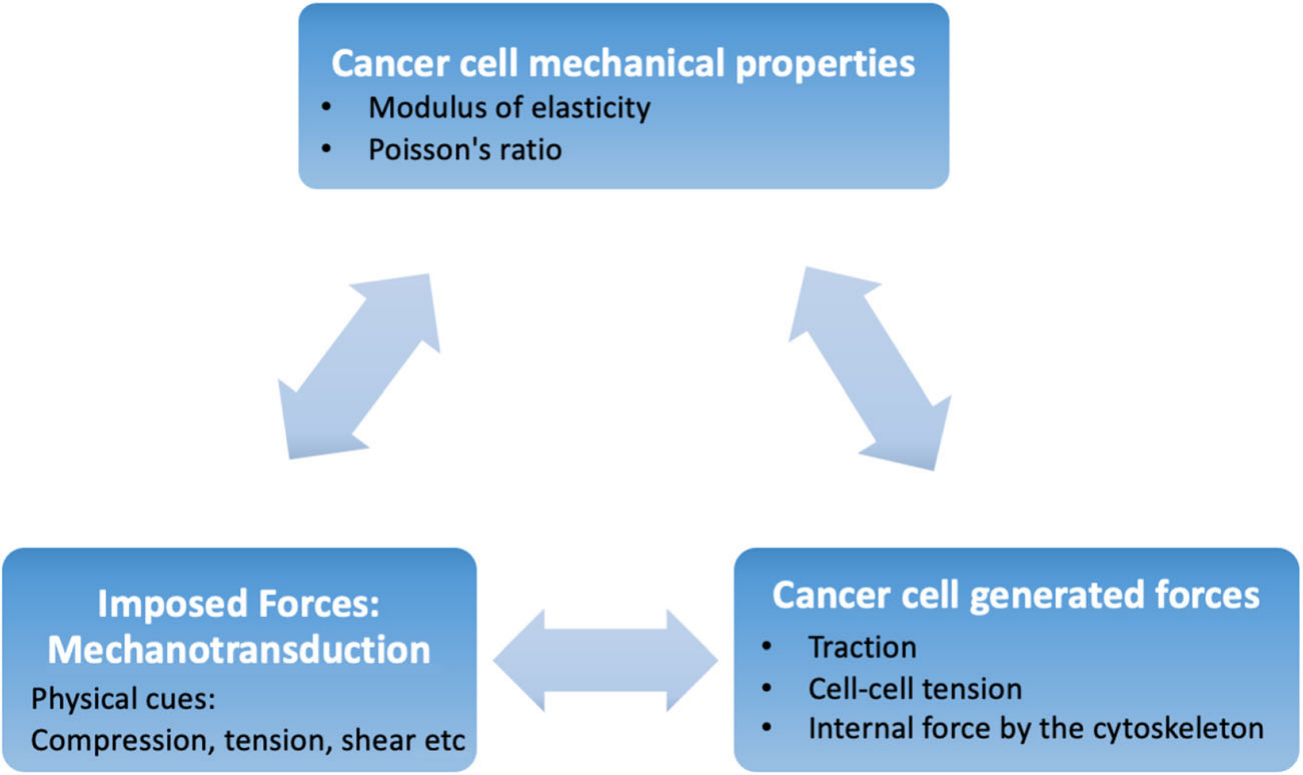
Mechanobiology of cancer cells is categorised into three subthemes: (a) cell mechanical properties, (b) imposed forces translated to biochemical signals through mechanotransduction, and (c) intracellular generated forces

One of the research areas gaining interest is the mechanobiology of cancer cells, which is broad in scope, so is commonly (and for the purposes of this review) categorized into three sub-themes. The first of these is *cancer cell mechanical properties*, where the modulus of elasticity and the Poisson’s ratio (of the cells as a whole and those of the individual organelles) are investigated, in order to shed light on cancer cell migration through the matrix. This theme contributes to knowledge of the mechanism by which cancer cells invade the stromal matrix and the vasculature and metastasize to other organs. The second theme, *mechanotransduction*, describes how physical cues trigger the beginning of signaling pathways within the cancer cell. This covers a wide range of events, from ion channel—or protein kinase—activation, to changes in cell-phenotype, which occur over the long term and require initiation of gene transcription, as well as protein production. The response of cancer cells to applied forces and shear stresses inside the microenvironment of the tumor are covered in the third theme *cancer cell-generated forces*. These forces are thought to play a critical role in cell adhesion, function, and signaling and have been hypothesized to affect both the growth of the tumor and metastasis to distant organs (Fig. 1).

Research in the area of cancer mechanobiology is of paramount importance in understanding the mechanism of metastasis. The metastatic process is initiated by tumor cells disrupting existing adhesion bonds between neighboring cells and disconnecting from the primary tumor Friedl and Wolf (2003). Subsequently, cancer cells travel inside the stroma by exerting forces and simultaneously degrading matrix fibers. Cancer cell deformation is a key feature of this process, as they literally squeeze to fit into the matrix pores, and then to pass through the walls of the blood or lymphatic vessels surrounding the primary tumor, a process called intravasation (Van Zijl et al. 2011; Deryugina and Quigley 2015). As they travel around in circulation, cancer cells have to withstand increased forces from the blood (Aceto et al. 2015). The final part of this cellular journey comes with the exodus of cancer cells from the vessels after they adhere to the wall’s lumen, a process called extravasation Reymond et al. (2013). From that point on, cancerous cells disseminate into proximal sites to form secondary tumors, by colonization. These metastatic tumors can be within vessels or in distant organs (Nguyen et al. 2009). A prerequisite for this process is that the tumor microenvironment becomes a dynamic landscape with multiple interactions among cancer cells, immune cells, stromal cells, and the extracellular matrix (ECM).

Metastasis would not be possible if cancer cells were not able to apply forces to neighboring cells and be able to be distorted and reshaped to accommodate their passing through densely woven tissues (Chin et al. 2016; Polacheck and Chen 2016; Lintz et al. 2017). Although malignant tumors start by genetic aberrations, the progression of cancer and its ability to metastasize depend largely on interactions between tumor cells, normal cells, and noncellular substances in proximity. These interactions are driven by both mechanical and biologic processes, which are often closely associated (Hanahan and Weinberg 2011; Pickup et al. 2014) (Table 1).

**Table 1:** Translation of mechanical cues into intracellular biochemical signaling promotes metastasis

Stimuli	Biochemical pathway	Mechanical changes	Result
Mechanical signals from the ECM Butcher et al. 2009 ➔ EMT	• Loss of E-cadherin expression • Increase of N-cadherin expression Yang and Weinberg 2008	• Reorganization of the actin cytoskeleton • Change of cell polarity • Cell shape change: regular columnar to irregular rounded or elongated • Mesenchymal-like phenotype through EMT Yang and Weinberg 2008	Weaker cell–cell adhesions ➔ dissociation of cells from the primary tumor (Northcott et al. 2018)
EMT**➔** Coordinated activation of intracellular protrusive and contractile forces (Tighe et al. 1989; Stucki et al. 2001; Suresh 2007)	Activation of Rho GTPases at the front➔ peripheral actin polymerization Yamaguchi and Condeelis 2007	Lamellipodial protrusions	• Lamellipodia result in attaching and pulling cell body • Cell elongation and directional motility (Lamuille et al. 2014)
EMT**➔** Coordinated activation of intracellular protrusive and contractile forces (Tighe et al. 1989; Stucki et al. 2001; Suresh 2007)	• Activation of Rho GTPases at the leading edge ➔ F-actin polymerization and depolymerization and actin-binding proteins (Buccione et al. 2004; Destaing et al. 2011) • ARP2/3 complex and its activators, i.e., NWASP WASF1, WASF2, WASF3) Yamauchi et al. (2005)	Invadopodia formation (Artym et al. 2006; Hall 1998; Wolf and Friedl 2009; Stevenson et al. 2012)	Matrix degradation and ECM destruction (Artym et al. 2006; Hall 1998; Wolf and Friedl 2009; Stevenson et al. 2012)
Mechanical signals from the ECM Butcher et al. 2009	Activation and oligomerization of integrins and mechanosensors (e.g., talin, vinculin), signaling molecules (e.g., FAK signaling) **(**Butcher et al. 2009; Yu et al. 2011) ➔ Forming the focal adhesion complex (FA) (Wozniak et al. 2004) ➔ ROCK activation at the rear	• Myosins associated with actin filaments form actin/myosin complexes➔ actomyosin stress fiber formation (Dube et al. 2016) • Increased actomyosin contraction➔more cell- cell detachment • Increased cell- ECM adhesions (Provenzano et al. 2009)	• Further contractility in feedback loop (Levental et al. 2009) and intracellular forces needed for migration (Suresh 2007; Janmey et al. 2016; Stricker et al. 2010) • Increased integrin matrix adhesion through FA • Enhanced invasive potential (Levental et al. 2009)
Stiff ECM ECM (Staunton et al. 2016)/ Cellular traction on ECM (rich in collagen I and fibronectin) (Hotary et al. 2003; Levental et al. 2009)	MMPs expression➔ digestion of laminin and collagen of ECM	Stress fiber formation	Translation through ECM
Stiff ECM (Staunton et al. 2016)/cellular traction on ECM (rich in collagen I and fibronectin) (Hotary et al. 2003; Levental et al. 2009)	MMPI expression ➔ inhibition of further MMPs expression (Kumar and Weaver 2009)	• Less-organized cytoskeleton➔ cytoplasm softening (Guck et al. 2005; Cross et al. 2007) • Decreased Young’s modulus in invasive cells (Faria et al. 2008; Lekka et al. 2012; Xu et al. 2012)	Cells “squeeze” (Kumar and Weaver 2009) ➔ invasion through ECM
Actomyosin contractility Gupta et al. 2012; Jain et al. 2013)	Changes in chromatin organization and LINC complexes (Crisp et al. 2006; Lee et al. 2007; Gerlitz and Bustin 2010) mediated by interactions between SUN domain-containing proteins (SUN1 and SUN2) and Klarsicht homology (KASH) domain-containing proteins (nesprin 2 and nesprin 3) (Starr and Han 2003; Technau and Roth 2008)	Cellular shape alterations, protrusive formations, nucleus’ elastic properties (Crisp et al. 2006; Lee et al. 2007; Gerlitz and Bustin 2010)	Signals are directly transduced to the nucleus through physical linking between the nuclear lamina and the cytoskeletal networks Crisp et al. (2006)
Bloodstream shear stress	increased activation of integrins and their receptors (ICAM, VCAM) for cell-EW binding (Strilic and Offermanns 2017; Rejniak 2007; Rejniak 2012; Berger et al. 2002; Luo et al. 2007) ➔ Src signaling pathway (Thamilselvan et al. 2007) and FAK phosphorylation Haier and Nicolson (2001)	• cell shape alterations • cortex stiffness alterations (either increase (Khismatullin 2009; Mofrad and Kamm 2006) or decrease (Stoletov et al. 2010a, 2010b; Yamauchi et al. 2005) depending on locomotion from floating till crawling) • Reorganization of the actin cytoskeleton and creation of more focal adhesions (Thamilselvan et al. 2007)	enhancement of cell to EW attachment (Haier and Nicolson 2001; Von Sengbusch et al. 2005)➔ early arrest (Zhu et al. 2008) and survival of CTCs ➔ extravasation through the formation of specific bonds (Follain et al. 2018; Stoletov et al. 2007; Chen et al. 2013)
Bloodstream shear stress	• P-selectin glycoprotein ligand 1 (PSGL1) or platelet endothelial adhesion molecule 1 (PECAM1) (Reymond et al. 2013; Frenette et al. 1995)	• Clusters with platelets: platelet-mediated capture (analogous to nucleation and growth)(Reymond et al. 2013; Frenette et al. 1995)	Enhance arrest (Reymond et al. 2013; Frenette et al. 1995)
Clusters with platelets	• Releasing bioactive agents like vascular endothelial growth factor (VEGF) in the endothelium (Felding-Habermann et al. 1996; Burdick and Konstantopoulos 2004; Gay and Felding-Habermann 2011)	• Increase in vascular permeability (Felding-Habermann et al. 1996; Burdick and Konstantopoulos 2004; Gay and Felding-Habermann 2011)	Extravasation of cancer cells (Felding-Habermann et al. 1996; Burdick and Konstantopoulos 2004; Gay and Felding-Habermann 2011)

*NWASP* neural Wiskott–Aldrich syndrome protein, *WASF1*, *WASF2*, *WASF3* Wiskott–Aldrich syndrome protein family; *ECM* extracellular matrix, *EMT* epithelial to mesenchymal transition, *FAK* focal adhesion kinase, *ROCK* Rho/Rho kinase, *ICAM* intercellular adhesion molecule 1, *VCAM* vascular cell adhesion molecule 1, *EW* endothelial wall

Lately, there is a growing interest in how the mechanical properties of metastatic cells are altered in relation to nonmetastatic or normal cells and the way these alterations affect the cell’s metastatic potential. Knowledge and understanding of the biophysical mechanisms that drive cancer progression is crucial, as it may be a helpful tool for both the improvement of cancer prevention and the development of novel therapeutic approaches in the treatment of cancer. Therefore, the purpose of this review was to study the mechanisms of metastasis from a biomechanical perspective and investigate the interactions of cancer and normal cells during this process. These mechanisms are influenced by the following: (i) alterations of intracellular mechanical properties, (ii) migration through ECM, (iii) nucleus compliance, and (iv) biologic fluids and their mechanical effect on metastasis.

### Alterations of intracellular mechanical properties

It is well documented that during progression of cancer, cells are exposed to mechanical forces from the dynamic tumor micro-environment. Cells need to adapt to the stresses and modify their mechanical properties in response to both mechanical and chemical stimulation, resulting in the activation of different signaling pathways through the process of mechanotransduction. The latter describes the translation of external mechanical cues (e.g., ECM rigidity, compression, tension) into chemical signals within the cell (Schwander et al. 2010; Northcott et al. 2018), which actually act as a mechanosensor (Butcher et al. 2009). The active interaction of cancer cells with this mechanically mediated microenvironment leads to changes of their intracellular mechanical properties. These alterations are mainly regulated by the cytoskeleton, a complex network of filamentous actin, microtubules, and intermediate filaments extending from the cell cortex to the nucleus (Suresh 2007; Fletcher and Mullins 2010; Pritchard et al. 2014; Jiang et al. 2021). The resulting changes to cytoskeletal structure and cellular shape can in turn affect adhesion, proliferation, differentiation, survival/death, and/or migration, all of which are linked to tumor progression and aggression (Butcher et al. 2009; Yu et al. 2011). This is a complex process, regulated to a certain extent by alteration of gene expression, which has been proven to play a critical role in cellular modifications driven by mechanical stimuli (Schmitz et al. 2000; Gupta et al. 2012; Jain et al. 2013; Jiang et al. 2021).

**Fig. 2:**
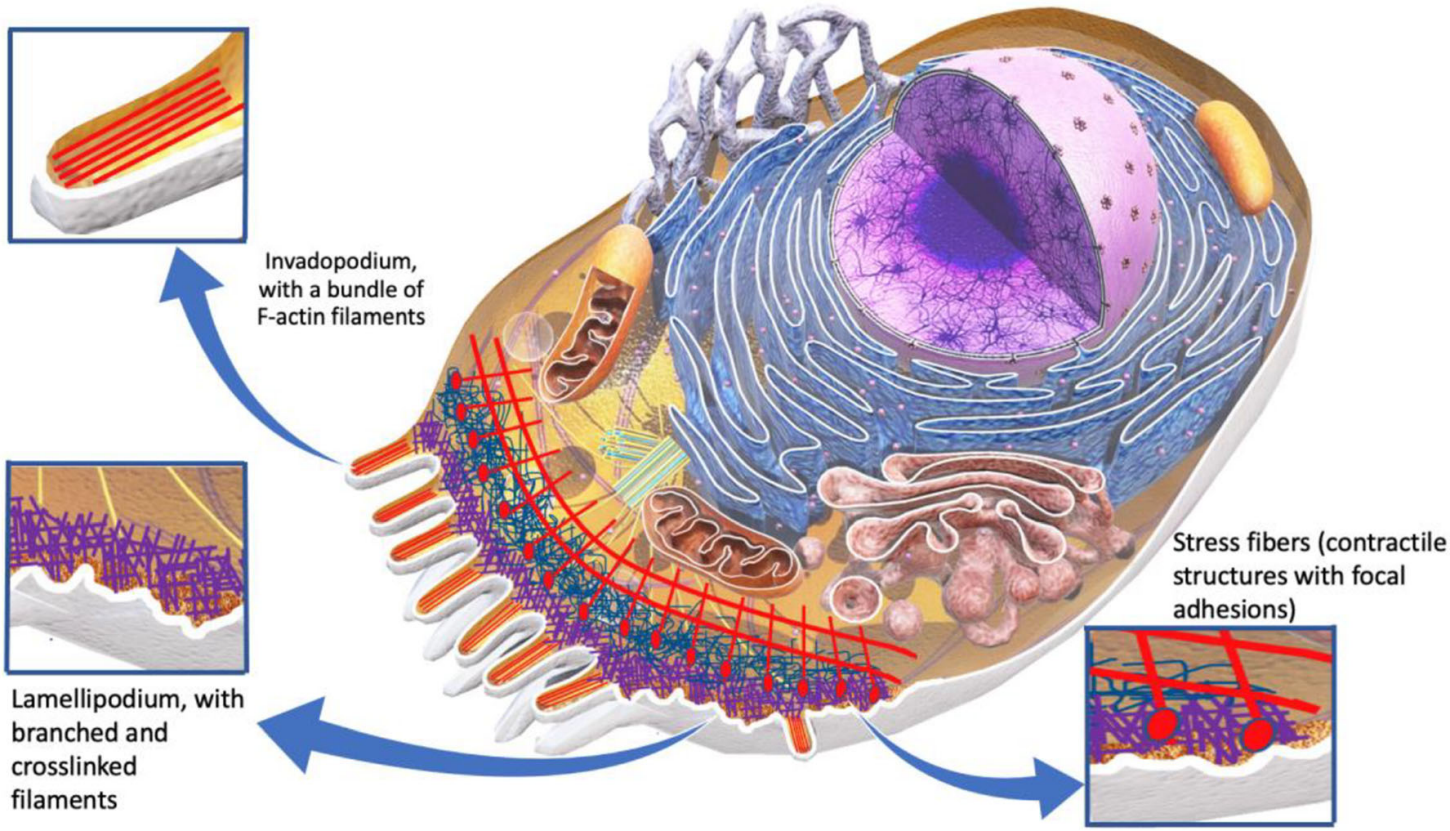
Tumor cell components and cellular protrusions. During migration, cells generate increased protrusive forces and form membrane protrusions, known as lamellipodial protrusions. These are flat broad protrusions at the leading edge created from peripheral actin polymerization. Tumor cells migrating through the rigid ECM also form invadind protrusions, called invadopodia. These are finger-like F-actin protrusions that have an ECM remodeling activity via matrix-degradation and ECM destruction

Cancer cell migration is a multistep dynamic process that requires biochemical and biophysical reorganization of cell–cell adhesions, cell–matrix adhesions, and alteration of cell shape or polarity, resulting from the coordinated activation of intracellular protrusive and contractile forces (Tighe et al. 1989; Stucki et al. 2001; Tallman et al. 2002; Suresh 2007). It has long been proven that a critical requirement of the metastatic process is the reorganization of the actin cytoskeleton. Actin is the primary component of the cytoskeleton, a main mediator for intercellular force generation and a key component for cell spreading and adhesion. Reorganization of the actin cytoskeleton is important for the transition of epithelial-like cells to mesenchymal-like cells through a well-characterized process, known as epithelial–mesenchymal transition (EMT) in embryogenesis (Kalluri and Weinberg 2009; Thiery et al. 2009; Morales et al. 2021). This is a crucial step in detachment of tumor cells from the epithelium and the invasion of the ECM. During EMT, malignant cells reorganize their actin formation in the cytoskeleton, which leads to a cell migratory phenotype characterized by cell elongation and directional motility Lamuille et al. (2014). This migratory potential presupposes a modification of the cell’s behavior which exhibits increased peripheral actin polymerization and finally results in an increase in protrusive forces and formation of protrusions, known as lamellipodial protrusions. These are flat broad membranous protrusions located at the leading edge of the migrating cells and are responsible for driving the migrating cells through actin filament polymerization (Yamaguchi and Condeelis 2007; Masi et al. 2020). Except from lamellipodia, invadopodia are also key components that command the direction of the migrating cells and contribute to cancer cell invasion via matrix-degradation and ECM destruction (Artym et al. 2006; Hall 1998; Bryce et al. 2005; Winder and Ayscough 2005; Yamauchi et al. 2005; Wolf and Friedl 2009; Stricker et al. 2010; Hall 2012; Stevenson et al. 2012; Masi et al. 2020). These are finger-like F-actin protrusions formed by cancer cells during migration. More specifically, F-actin is subjected to rapid polymerization and depolymerization through the activation of Rho GTPases and actin-binding proteins (Buccione et al. 2004; Destaing et al. 2011) (Fig. 2).

Although the structure and molecular composition of invadopodia remain under investigation by the research community, it has been demonstrated that the formation of these structures requires highly localized actin polymerization and coordinated action of multiple binding proteins, including those that constitute the F-actin nucleating ARP2/3 complex and its activators, i.e., neural Wiskott–Aldrich syndrome protein (NWASP) and Wiskott–Aldrich syndrome protein family (WASF1,WASF2, WASF3) (Yamaguchi et al. 2005a, 2005b; Masi et al. 2020; Jiang et al. 2021). Hence, in the leading edge of the migrating cells, there are lamellipodial protrusions and localized invadopodia, which generate protrusive forces by localized actin polymerization at the plasma membrane, as observed in studies using AFM to measure the force exerted by actin cytoskeleton (Chien et al. 1987). Actin polymerization is the leading mechanism that creates protrusive forces in cells and plays a very significant role to cell migration (Bryce et al. 2005). These cell protrusions lead to a rapid increase in cell area and are commonly the first connecting areas between the migrating cells and the ECM (Yamaguchi and Condeelis 2007). An increase in cell surface area provides an increase in the number of focal adhesions, too. Protrusions serve as both anchoring points for the cell adhesion to the matrix and migration, and as mediators in transmission of information from the ECM (Yamaguchi and Condeelis 2007).

Adhesion molecules associated with the formation of polarized actin bundles create a dynamic connection between the cellular actin network and the ECM fibers, which play a critical role in reciprocating forces between cells and the surrounding environment (Ghosh and Dawson 2018). It has also been shown that mechanical signals from the ECM provoke the activation and oligomerization of integrins, which are heterodimeric transmembrane adhesion proteins that can act as force sensors for cells (Butcher et al. 2009). After the activation and increase of integrin adhesions, their maturation into focal adhesions has been noted (Butcher et al. 2009; Harris et al. 2018). The extracellular part of an integrin interacts with matrix proteins, including collagen and fibronectin, while the intracellular part recruits focal adhesion proteins, including mechanosensors (e.g., talin, vinculin), signaling molecules (e.g., focal adhesion kinase), helping to form the focal adhesion complex (FA) and actin binding proteins (e.g., filamin, a-actinin) (Morales et al. 2021). All these contribute to the connection between integrins and the cytoskeleton (Wozniak et al. 2004).

Actin can generate forces both through its localized polymerization and through coupling to its associated motor protein, myosin. Protrusions at the leading edge are formed as a result of localized actin polymerization, whereas retraction of the trailing edge is controlled by contractile forces generated by myosin motors (Ridley et al. 2003; Chi et al. 2014). Myosins are included in the category of motor proteins which, when associated with actin filaments form actin/myosin complexes and generate the cellular forces used in cell contractility and migration. Cells are constantly receiving and responding to the mechanical stimuli of the environment by a compensatory expression of the actin-bundling protein tropomyosin, which correlates with actomyosin stress fiber formation (Dube et al. 2016). It has been proven that this protein has a critical role in cell motility and stiffness, as it provides contractile forces inside the cell. These forces are generated through intracellular tension, as a result of myosin II activity and contractility and are essential for the controlled detachment of the rear of a cell from the substratum and enhancement of actin polymerization at the leading front part of the cell (Deree et al. 2006; Elkhatib et al. 2014; Fukumoto et al. 2015; Jalilian et al. 2015; Morales et al. 2021).

**Fig. 3:**
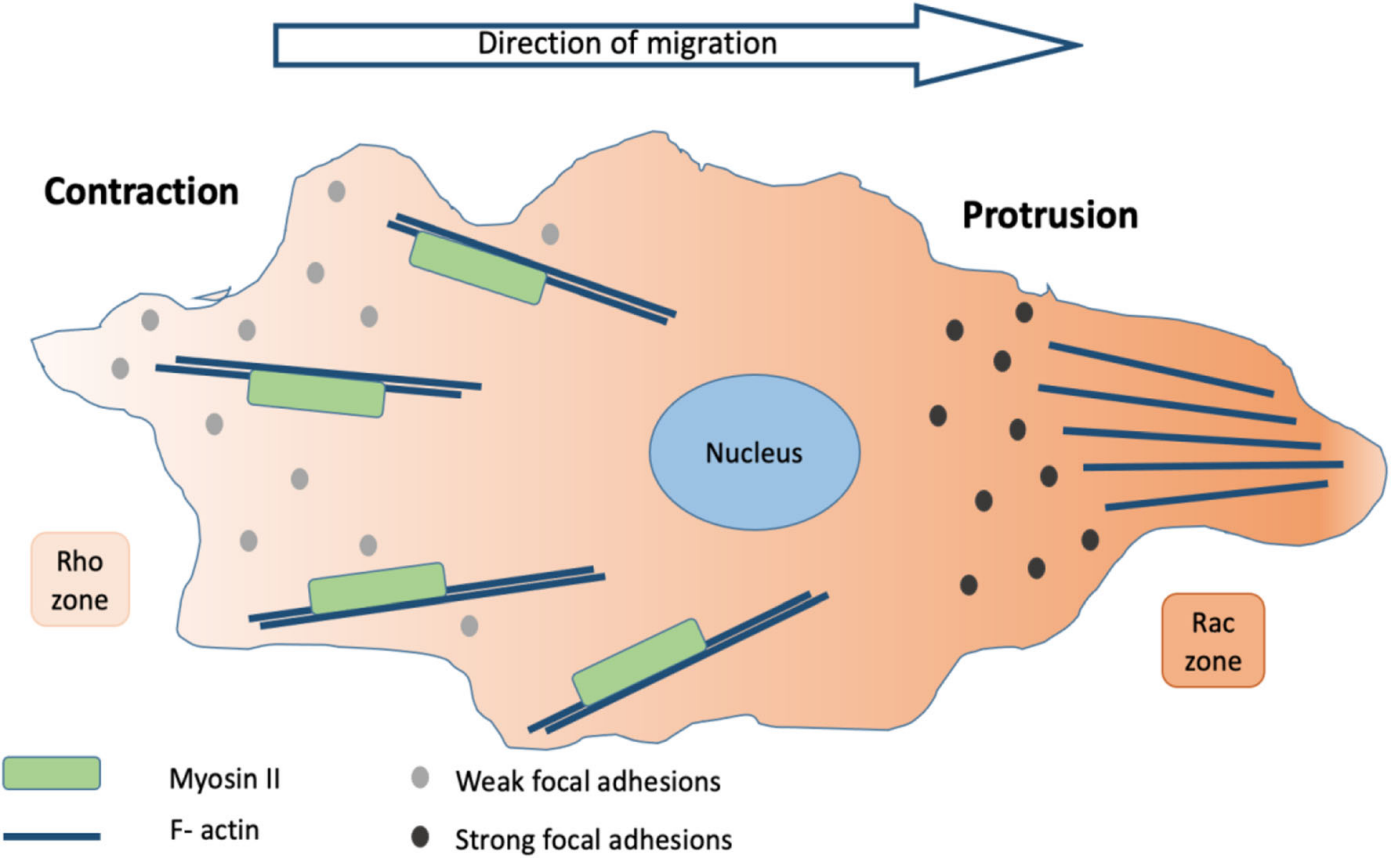
Tumor cell shape and polarity alterations drive the cytoskeleton. The interaction of cancer cells with the mechanically mediated microenvironment leads to changes of their intracellular mechanical properties. These alterations are mainly regulated by the cytoskeleton, a complex network of filaments extending from the cell cortex to the nucleus which contributes to cell shape or polarity alterations. Actin, the main kind of filaments, can generate forces both through its localized polymerization and reorganization and through coupling to its associated motor protein, myosin. Protrusions at the leading edge are formed as a result of actin localized polymerization, whereas retraction of the trailing edge is controlled by contractile forces generated by myosin motors. All of these cellular responses are followed by modified gene expression, which is described as a localized Rac activation at the front and Rho activation at the rear

**Fig. 4:**
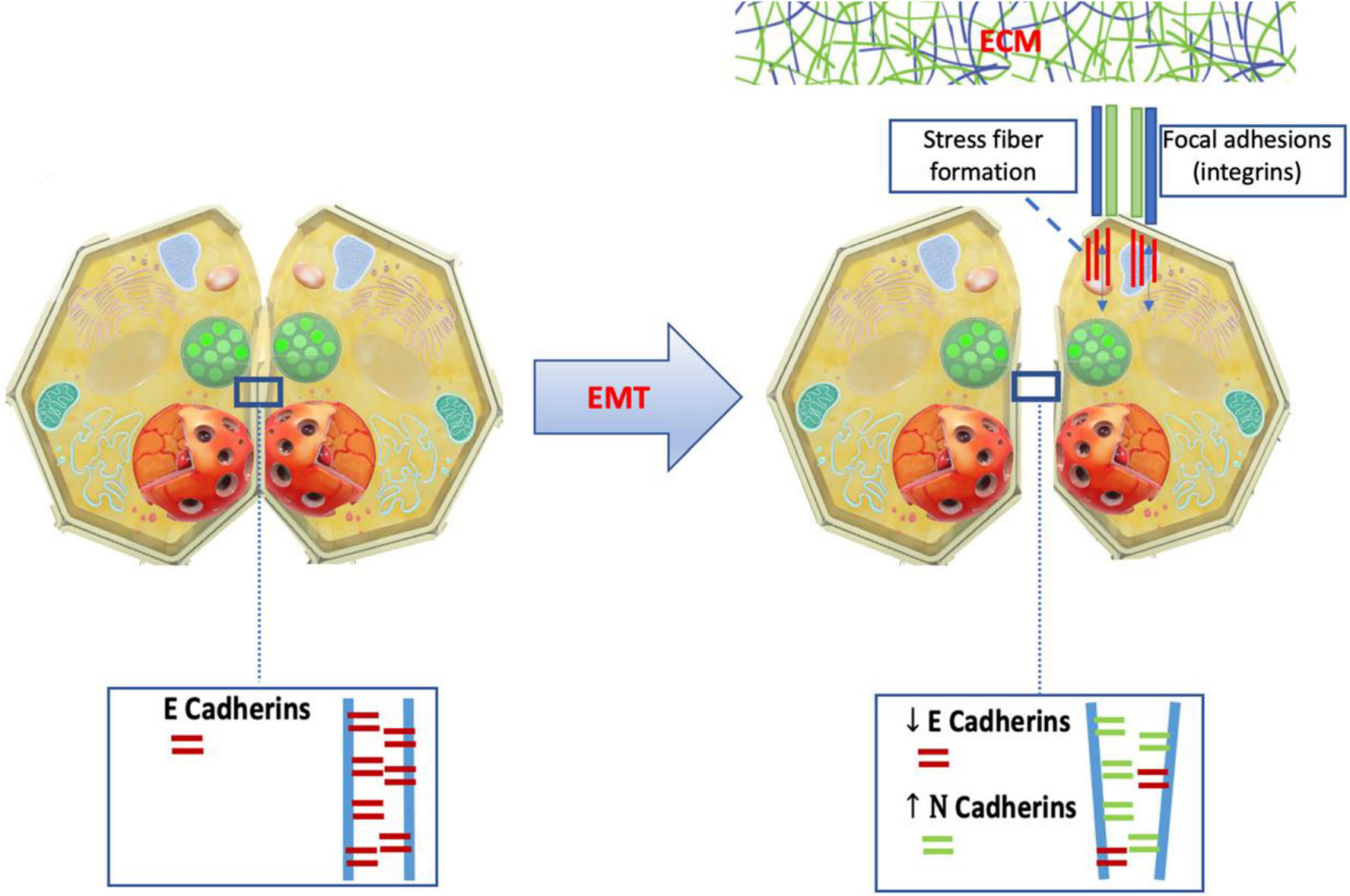
Epithelial to mesenchymal-like cell transition (EMT). Cancer cell migration requires reorganization of cell–cell adhesions and cell–matrix adhesions due to the intracellular protrusive and contractile forces. Cells can sense extracellular mechanical stimuli through force sensor proteins (integrins) that activate intracellular signaling pathways. Integrin dimerization results to the maturation of focal adhesion complex and actin polymerization producing intracellular tension through stress fiber formation. Cells that undergo epithelial to mesenchymal transition (EMT) exhibit an alteration in adherens junction proteins (i.e., cadherins). A loss of E-cadherin expression in favor of N-cadherin expression takes place. This leads to weaker cell–cell adhesions and formation of a cell with increased motility

It is well established that cell migration, as a dynamic process, occurs through the coordinated alterations of intracellular contractile and protrusive forces (Tighe et al. 1989; Stucki et al. 2001; Tallman et al. 2002; Suresh 2007). In general, it is widely accepted that actin polymerization at the front side of the cell promotes the protrusive activity via invadopodia formation. This activity, in combination with actomyosin filaments that create contraction at the sides and posterior part of the cell, form the main intracellular forces needed for migration (Suresh 2007; Stricker et al. 2010; Janmey et al. 2016). All of these cellular responses are directly connected to modified gene expression, which is described as a localized Rac activation at the front and Rho activation at the rear. It should be mentioned however that, this process is not completely clear, and different cell types move in different ways (Fig. 3). Nevertheless, it suggests a possible mediator for migration (Schmitz et al. 2000; Pertz et al. 2006). In fact, except from the actin bundles reorganization, another major step in tumor metastasis is the isolation of a single cell from the primary tumor and its invasion to the ECM. This detachment of tumor cells from the epithelium and the invasion of the ECM is quite similar to the well-described transition from epithelial to mesenchymal (EMT) in embryogenesis (Kalluri and Weinberg 2009). It is well established that increased intracellular tension through actomyosin contractility produce morphogenetic changes in epithelial cells during the invasive progression. Cells that undergo EMT exhibit an alteration in adherens junction proteins, which are complexes transmitting intercellular tension and are composed of receptors (i.e., cadherins), mechanosensors (i.e., catenins), linker proteins, and signaling molecules (i.e., SRC). The cadherin family of proteins plays a critical role in the dissociation of cells from the primary tumor; these are glycoproteins with a single transmembrane domain and are the major mediators of intracellular adhesion (Northcott et al. 2018). Specifically, it has been shown that a loss of E-cadherin expression in favor of N-cadherin expression takes place. This has proved to create weaker cell–cell adhesions and acquisition of a mesenchymal-like phenotype with increased motility (Yang and Weinberg 2008) (Fig. 4).

### Migration through ECM

The processes described above stimulate formation of cells with less intracellular connections and stronger attachment with the ECM. In cases of a stiff ECM, like the one found in tumor sites, a focal adhesion mediator, i.e., talin mechanosensory, is activated and transmits the signal from the ECM to the cell through stimulation of a FAK signaling molecule (Butcher et al. 2009; Yu et al. 2011). Focal adhesion formation and further maturation results in Rho/Rho kinase (ROCK) activation and strengthens actomyosin contraction and the resulting cell–cell detachment.

Besides the increase in the number of focal adhesions resulting in a stronger attachment of the cell to the ECM, cells also begin to express matrix metalloproteinases (MMPs) on their surface. These molecules lead to the disruption and digestion of the laminin and collagen IV present in the basement membrane. By the time the invasive tumor cells leave the primary tumor, they enter the complex ECM, which is rich in collagen I and fibronectin. These components often make it stiffer than normal tissue, due to the increased collagen deposition (Hotary et al. 2003; Levental et al. 2009; Kai et al. 2019; Amos and Choi 2021). Collagen I, specifically, has been found to increase local stiffness up to 50 times (Liu and Cao 2016). In addition, collagen crosslinking reinforces integrin expression, resulting in the formation of even more cell–ECM adhesions (Provenzano et al. 2009; Kai et al. 2019). Such changes of the matrix can further enhance the invasion potential and cause further cell contractility in a positive feedback loop (Levental et al. 2009).

Remodeling of the existing matrix, secretion of new matrix, as well as inhibition of MMP activity, force the cells to undergo cytoskeletal and nuclear deformations. In this way cells can “squeeze” through the collagen fibers and support subsequent cell invasion (Kumar and Weaver 2009). More specifically, cell compliance was observed to be tuned by the extracellular matrix, due to increased actomyosin contractility, caused as tumor cells invade into ECM collagen fibers (Staunton et al. 2016; Kai et al. 2019). Biophysical measurements comparing the mechanical responses of normal and cancer cells have proven that cancer cells seem to become more compliant than their normal counterparts. The increased deformability of malignant cells is directly related with an increased metastatic potential (Tian et al. 2020). In cancer cells, a softer cytoplasm correlates with a less-organized cytoskeleton (Guck et al. 2005; Cross et al. 2007). More specifically, the Young’s modulus has been found to decrease when cells become invasive, compared to normal ones (Faria et al. 2008; Lekka et al. 2012; Xu et al. 2012). These elasticity changes are often falsely recorded, due to the tumor’s stiffer environment, as observed recently by combining AFM and confocal microscopy (Prabhune et al. 2012). Indeed, it has been demonstrated in the past that most solid tumors appear to be stiffer than healthy tissues, possibly due to the increased tumor ECM stiffness (Paszek et al. 2005; Butcher et al. 2009). It should be noted however that cancer cells per se present a lower stiffness compared to normal cells, and therefore are more deformable (Guck et al. 2005; Cross et al. 2007). In fact, research on breast cancer cells has shown that the metastatic potential of malignant cells is associated with their deformability properties (Guck et al. 2005). Furthermore, Cross and colleagues, using atomic force microscopy (AFM) to examine cell compliance, showed that patients’ normal cells are stiffer than their tumor cells (Cross et al. 2007). Several other studies have also demonstrated that cancer cells display increased deformability and compliance, compared to normal cells, due to alterations in biochemical processes (Cross et al. 2008; Ahn et al. 2010; Lee et al. 2012). One of those studies actually suggests a ranking order of Young’s modulus at 1 nN stiffness of indentation force, measured with the AFM method. Normal squamous cells (EPC2) present a Young’s modulus of 4.7 kPa when alive and 9.9 kPa when fixed, and seem to be stiffer than metastatic cells (CP-A), which present corresponding values of 3.1 kPa and 2.9 kPa. Metastatic cells still had greater elastic moduli than dysmoplastic esophageal cells, which presented values of 2.6 kPa (live) and 2.1 kPa (fixed) (Ahn et al. 2010).

The mechanical properties of cancer cells change in response to the mechanical forces, demonstrating that cells can actively become stiffer, by actin reorganization and polymerization (Icard-Arcizet et al. 2008). It should be mentioned that apart from the physical forces mentioned earlier, mechanical forces are exerted to cancer cells also through the interstitial flow, due to the slow fluid movement within the ECM. Interstitial flow refers mainly to lymphatic drainage, a process with which plasma returns to bloodstream. This process is governed by the Starling equation (Eq. 1) (Woodcock and Woodcock 2012):

1 $$ {J}_v={L}_pS\left(\left[{P}_c-{P}_i\right]-\sigma \left[{\pi}_p-{\pi}_g\right]\right) $$

where *J*_*v*_ is the trans-endothelial solvent filtration volume/s, *L*_*p*_ is the membrane’s hydraulic conductivity, *S* is the available filtration area, *P*_*c*_ is the hydrostatic pressure in the capillary, *P*_*i*_ is the interstitial hydrostatic pressure, *σ* is the Staverman’s reflection coefficient, *π*_*p*_ is the plasma protein oncotic pressure, and *π*_*g*_ is the subglycocalyx oncotic pressure.

It has been shown in the past that a significant elevation of interstitial flow can occur in the microenvironment of the tumor, which can affect crucially the progress of the disease (Chary and Jain 1989). Experiments in mice have demonstrated that increased interstitial flows are present in the tumor microenvironment, probably due to elevated neoplastic interstitial fluid pressure (IFP) (Boucher and Jain 1992). This finding directly affects tumor angiogenesis, due to the increased mechanical forces applied to the ECM (Boucher et al. 1996).

Certain biological factors and processes are also affected by the IFP, which causes lymphatic vessels to upregulate expression of adhesion molecules such as ICAM-1 and E-selectin and of chemokines such as CCL21 (Miteva et al. 2010). These events result in an enhanced metastatic potential, as cancer cells are directed toward lymphatic vessels, and transmigration into the lymphatic vessels has been observed (Johnson et al. 2006; Shields et al. 2007; Miteva et al. 2010).

### Nucleus compliance

Although the common perception is that the cell, as a whole, needs to be softer to allow more sufficient migration, a recent study demonstrated that greater nucleus compliance predisposes for metastatic activity (Mekhdjian et al. 2017). The nucleus is known as one of the most important organelles in normal cells, due to the fact that it contains the genetic material (DNA). The nucleus also plays an important structural role inside the cell, as it occupies the largest space inside the cytoplasm and has been found to be approximately ten times stiffer than the cytoplasm (Dahl et al. 2004; Tseng et al. 2004; Friedl et al. 2010). As a result, nuclear mechanical properties could limit the cells’ ability to penetrate the dense matrix Friedl and Alexander (2011). For example, if the nucleus cannot squeeze through a pore, then the cell becomes unable to invade unless the matrix is highly degraded (Janmey et al. 2016).

Recent data advocate that the nucleus is not only important in migration due to its deformability but also because of its connection to the cytoskeleton (Wirtz 2009). The nucleus connects to the cytoskeleton through the LINC complex and recent data suggest that this connection plays a critical role in pseudopodial extension during 3D migration (Khatau et al. 2012). Therefore, it seems that nuclear deformity makes the metastatic cell more compliant and allows it to navigate through solid ECM spaces (Lautscham et al. 2015). It should be mentioned however, that the nuclear mechanics of cancer cells is not a well-studied area (Janmey et al. 2016). Nevertheless, research has shown that components of the nuclear envelope and the nuclear lamina can determine the nucleus’ elastic properties through chromatin organization and LINC complexes (Crisp et al. 2006; Lee et al. 2007; Hale et al. 2008; Stewart-Hutchinson et al. 2008; Gerlitz and Bustin 2010). LINC complexes are protein assemblies that cover the nuclear envelope, acting as physical linkers between the nuclear lamina and the cytoskeleton (Crisp et al. 2006). These linking complexes are mediated by interactions between SUN domain-containing proteins (such as SUN1 and SUN2) and Klarsicht homology (KASH) domain-containing proteins at the outer nuclear membrane, including the nesprin 2 and nesprin 3, which can enchain actin bundles (Starr et al. 2001; Starr and Han 2003; Technau and Roth 2008). Hence, the impact of nuclear properties in cell deformation extends beyond the fact it determines cellular shape to a large extent. Another important factor is that actomyosin contractility can lead to nuclear deformations, which play a critical role in altering gene expression (Gupta et al. 2012; Jain et al. 2013). These data suggest that cell shape and nuclear deformations occurring during metastatic invasion result in altered global acetylation and can affect transcriptional processes (Janmey et al. 2016).

Reduction of LINC complex components, nesprins, and SUN proteins, for example, can cause nuclear shape alterations and lead to a softer and more compliant nucleus and cytoplasm (Lammerding et al. 2004). Thus, the invasive potential of cells through ECM is suggested to depend on adhesiveness, nuclear volume, contractility and, to a lesser extent, the cortical cell stiffness (Lautscham et al. 2015).

### Biologic fluids and their mechanical effect on metastasis

**Fig. 5:**
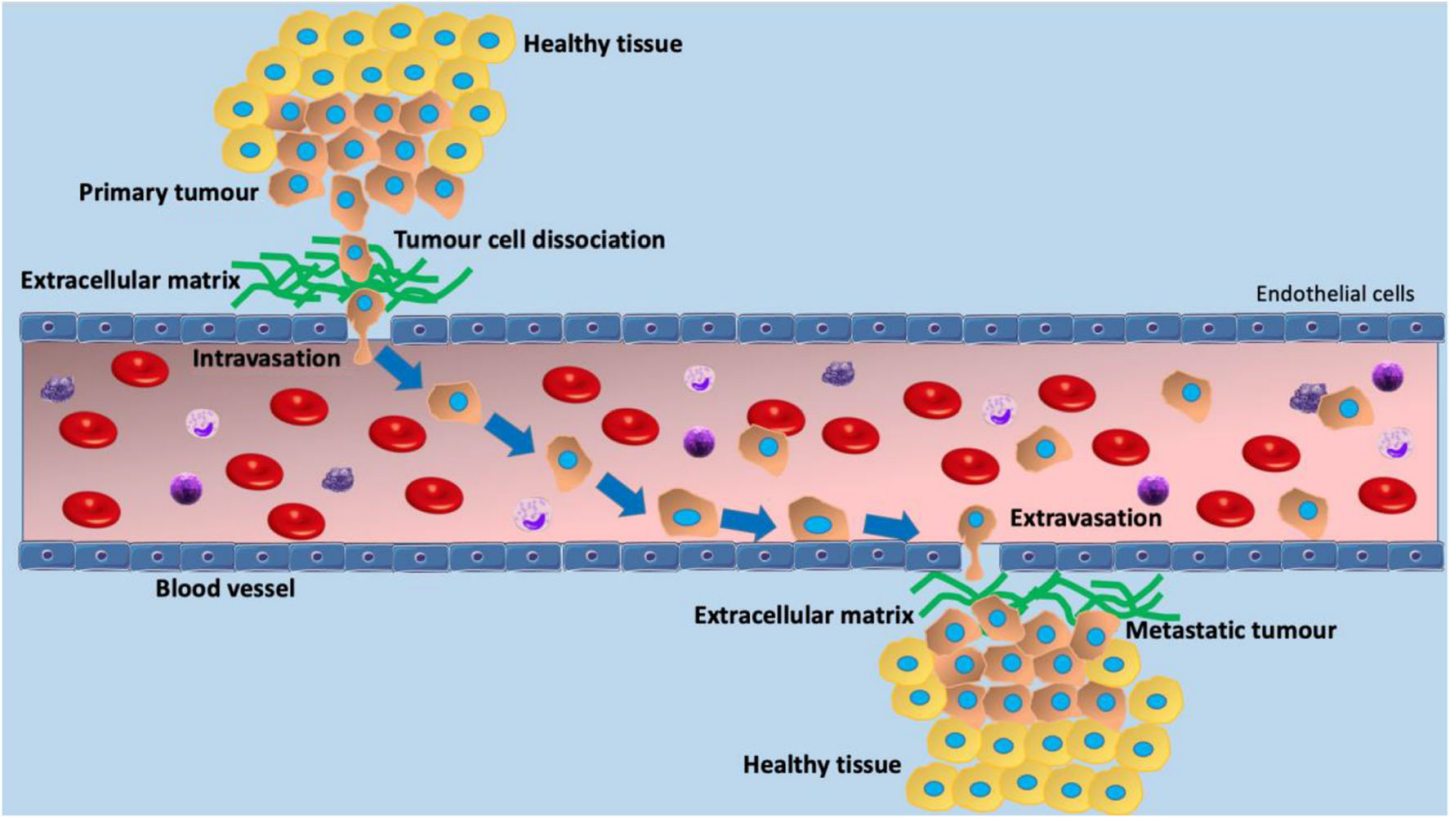
Metastatic process. In the metastatic process, cells detach from a primary tumor, penetrate the surrounding tissue, enter nearby blood vessels (intravasation). Since tumor cells manage to enter into the vascular system, their arrest and adhesion to the endothelium is an essential feature preceding their extravasation. Some of these cells eventually adhere to blood vessel walls and are able to extravasate and migrate into the local tissue, where they can form a secondary metastatic tumor. For a circulating tumor cell to enter or exit the circulatory system and migrate through it, it must adhere to the lumen of the vessel wall and squeeze through the EW cells

During the metastatic process, cancerous cells dislocate from the primary tumor and enter into the vasculature or lymphatic system through a process called intravasation (Quigley and Armstrong 1998; Friedl and Alexander 2011). Malignant cells then exit from blood vessels at secondary sites through extravasation. Important factors affecting these processes, as well as the survival of circulating tumor cells (CTCs) in the blood flow, include (i) flow rates, (ii) vessel diameter, and (iii) shear stress. Except from the multiple biological interactions driving metastasis, several studies suggest that biomechanical forces from fluids also contribute to tumor progression (Northey et al. 2017; Mohammadi and Sahai 2018; Martin et al. 2019). However, little research has been done related to the mechanics dealing with cancer cells’ intravasation and extravasation (Fig. 5).

In what follows we look more closely at a range of mechanical effects exerted by biological fluids on the CTCs within them:

#### Shear stress and early dissemination

When a tumor cell successfully migrates away of the primary tissue, two spreading routes are possible. Some cells manage to directly reach the blood vasculature, whereas others pass through the lymphatic system (Aceto et al. 2015; Follain et al. 2020). It is still unknown whether fluid biomechanics can influence the choice of early dissemination route of the tumor cell. It has been shown however that flow velocities and shear stress have lower rates in lymphatic than in blood circulation, as demonstrated in rats (Dixon et al. 2006). Lymphatic vessels are characterized mostly by laminar flow, pulsatile with low amplitude, and low velocities (Dixon et al. 2006). By contrast, blood has a much higher density of circulating components mostly consisting of blood cells and is characterized by higher flow velocities, due to cardiac output. In addition, blood flow can be pulsatile with high amplitude, whereas in veins, the flow is mostly laminar (Peng et al. 2017). Taking these into consideration, it is possible that passing through lymphatic vessels might, at early stages, be less disadvantageous to CTCs, rather than dissemination through blood flow (Dixon et al. 2006).

It is known that shear stress (*τ*) occurs between adjacent layers of fluid moving at different velocities. The velocity of a fluid in a cylindrical tube, like a vessel, seems to be at the maximum at the central parts and minimum, reaching zero, at the walls. The relative velocities of parallel adjacent layers of fluid in laminar flow determine the shear rate, which is defined as the increase of the velocity of the blood flow of two neighboring streaming blood layers. A definition of shear stress is given as the product of fluid viscosity and shear rate, and is expressed as units of force per unit area (N/m^2^ or dyn/cm^2^). The viscosity of blood has been calculated to be about 4 centipoise (cP), which is greater than the viscosity of water, which is 0.7 cP, at 37 °C. This increased viscosity is due to the presence of blood cells and components, primarily consisting of red blood cells. The normal time-averaged levels of shear stress vary between 1 and 4 dyn/cm^2^ in the venous circulation and 4 and 30 dyn/cm^2^ in the arterial circulation (Turitto 1982). The maximum shear stress appears near the vessel wall. The mean blood velocity (*v*_av_) in arteries for a vessel of 4 mm diameter is 0.45 m s^−1^, whereas the *v*_av_ for a 5-mm-diameter vein is 0.1 m s^−1^. The shear rates (d*γ*/d*t* = 8v_av_/*d*) are 900 s^−1^ in arteries, and 160 s^−1^ in veins, while shear rate values range from ~ 10 s^–1^ in the lymph (Dixon et al. 2006).

Shortly after reaching either the vasculature or the lymphatic system during metastasis, a tumor cell has to cope with a totally different set of mechanical forces, mainly imposed by fluid flow and shear stress (Kumar and Weaver 2009). The perception that fluid mechanics can affect the metastatic potential is based on the ‘hemodynamic theory’. This theory was suggested in a pivotal study, which supported a positive correlation between arterial blood flow and the frequency or the pattern of metastasis, concluding that a strong relationship exists between fluid mechanics and shear stress with the secondary metastatic tissue (Weiss et al. 1981). Yet, little is known about the effects of shear flow on the viability of CTCs. In addition, the influence of fluid shear to adhesive potential of CTCs to the endothelial wall (EW) remains to be determined (Wirtz et al. 2011).

#### Shear forces and CTC’s survival

It has been observed that once tumor cells manage to enter into the vascular system, they circulate in the bloodstream for only a limited period of time, which has not yet been determined precisely (Chambers et al. 2002). The deformation that CTCs experience in the bloodstream has been modelled by Rejniak and is governed by the following equations (Eq. 2–6) (Rejniak 2012):

2 $$ \rho \left(\frac{\partial u\left(x,t\right)}{\partial t}+\left(u\left(x,t\right)\cdotp \nabla \right)u\left(x,t\right)\right)=-\nabla p\left(x,t\right)+\mu \Delta  u\left(x,t\right)+{f}^{\ast}\left(x,t\right) $$

Equation 2 represents the Navier–Stokes equation for incompressible viscous fluids. It is expressed on the Cartesian system *x* = (*x*_1_, *x*_2_), *t* is the time, *f* is the external force density, while *ρ*, *u*, *p*, and *μ* represent the density, velocity, pressure, and viscosity of the fluid, respectively.

3 $$ \rho \nabla \cdotp u\left(x,t\right)=0 $$

Equation 3 represents the law of mass balance.

4 $$ f\left(x,t\right)={\int}_{\varGamma_t\cup {\varGamma}_e}F\left(l,t\right)\ \delta \left(x-X\left(l,t\right)\right) dl\kern0.75em $$

5 $$ \frac{\partial X}{\partial t}\left(l,t\right)=u\left(x,t\right){=\int}_{\varOmega}\mathrm{u}\left(x,t\right)\ \delta\ \left(x-X\left(l,t\right)\right)\  dx $$

Equations (4) and (Bell 1979) define the interactions between points X(*l,t*), which are on the cancer cell and the EW boundaries *Γ*_*e*_, *l* represents an index which is on the EW or on the cancer cell *Γ*_*t*_. In these equations, *F*(*l,t*) represents the force density acting on the EW and cancer cells and is applied to the fluid, using the two-dimensional Dirac *δ* function. *X*(*l,t*) represents all material boundary points which are transported with the fluid, while the boundary forces *F*(*l,t*) arise from EW rigid properties, cancer cell elastic properties, and adhesion from adhesion between the EW and from CTCs.

6 $$ F\left(l,t\right)=S\frac{\left\Vert X\left(l,t\right)-{X}^{\ast}\Big(l,t\Big)\right\Vert -L}{\left\Vert X\left(l,t\right)-{X}^{\ast}\Big(l,t\Big)\right\Vert}\left(X\left(l,t\right)-{X}^{\ast}\left(l,t\right)\right),\kern0.75em \mathrm{if}\ \left\Vert X\left(l,t\right)-{X}^{\ast}\Big(l,t\Big)\right\Vert \le {L}_{max}\kern1em $$

Equation 6 represents the short linear Hookean springs, in which *S* is the stiffness of the springs, *L* is the resting length of the spring, while X*(*l,t*) represents the opposite or adjacent point for, elastic, rigid, or adhesive forces, respectively.

There is evidence to suggest that intravascular death can be induced due to the mechanical forces from fluid shear stress (Weiss 1992; Wong et al. 2001). Most of the cancer cells entering the bloodstream are trapped in the vessels and are usually damaged in the microvasculature, a fact that ultimately makes the metastatic procedure inefficient (Weiss 1990). In fact, in vitro studies showed that less than 4% of cancer cells managed to form micrometastatic foci, after injection of tumor cells into vessels of metastatic organs. That research has also demonstrated that ~ 80% of the injected cancer cells bound to the vascular wall and went into arrest 1 day after injection (Luzzi et al. 1998; Cameron et al. 2000). In mouse brain metastasis, only 40–60% of the arrested cells retain blood flow forces and extravasate, suggesting ineffectiveness of the first metastatic steps (Kienast et al. 2010). It is assumed that CTCs suffer from shear stress that can cause cell cycle disruption. In vitro studies have shown that this can be caused by a shear stress of 12 dyn/cm^2^ (Chang et al. 2008), while cell structure damage and necrosis can be caused by shear values in the range of 6 dyn/cm^2^ (Mitchell et al. 2015; Regmi et al. 2018). In addition, apoptosis can be triggered by shear values of only 2 dyn/cm^2^ (Mitchell and King 2013). It is therefore concluded that even very low shear stress may render the metastatic procedure inefficient. It is interesting, however, that an oscillatory shear stress in the range of 4 dyn/cm^2^ did not lead to destruction or death of human tumor cells. This observation suggests that the cell type could play a key role (Lien et al. 2013). For example, CTCs were found capable of surviving high shear values, in the range of 60 dyn/cm^2^ for hours, but the effect on their metastatic and invasive potential remains to be investigated (Regmi et al. 2017).

#### CTC’s intravascular arrest

Upon successful intravasation, arrest and adhesion of CTCs to the endothelium are essential next steps preceding their extravasation (Reymond et al. 2013). Both in vitro and in vivo studies have described two main procedures responsible for the intravascular arrest of single or clustered CTCs (Follain et al. 2018; 2020). When the circulating tumor cell manages to enter to vessels with a diameter smaller than that of the cell, i.e., microvessels or capillaries, a process like mechanical trapping, called physical occlusion, occurs. Alternatively, when the cancer cell enters large blood vessels, the extravasation of the malignant cell requires its active adhesion to the vessel wall, through the formation of specific bonds (Stoletov et al. 2007; Chen et al. 2013; Follain et al. 2018). The ability to arrest depends on the binding potential between receptors on the circulating cell’s membrane and endothelial ligands (Zhu et al. 2008). It has been shown that sometimes physical occlusion is not sufficient for the arrest and extravasation. Therefore, active adhesion between CTCs and the vascular wall in combination with the mechanical trapping of CTCs is required for successful metastasis (Gassmann et al. 2009).

#### Fluid forces permitting stable adhesion of CTCs to the endothelium

Upon arrest, the collision between a tumor cell and the vessel wall may lead to transient or persistent adhesion, as a result of ligand–receptor interactions. Tumor cells have a finite adhesion force to the endothelial cells of the vessel wall, determined by the strength of the ligand–receptor adhesion pairs, and the forces exerted on the cells. Based on the simplifying assumptions that tumor cells have only one receptor class for ligand binding (Hammer and Lauffenburger 1987), that there is a homogeneous distribution of complexes in the contact area, and that ligand density (N_L_) remains nearly constant, as it is much larger than the receptor density (N_R_), Cozens-Roberts et al. presented a deterministic conservation equation for the reaction between the cell surface’s receptors and the immobilized ligand (Cozens-Roberts et al. 1990):

7 $$ \frac{dC}{dt_a}={k}_f^0{N}_L\left({R}_T-C\right)-{k}_r^0C $$

where *C* represents the receptor–ligand complexes number, *t*_*a*_ represents the attachment time, ${k}_f^0$ is the forward rate constant, *N*_*L*_ is the ligand density, *R*_*T*_ represents the total number of receptors which are available for binding within the contact area between the cell and the ligand, and ${k}_r^0$ is the reverse rate constant.

In order to overcome some inaccuracies associated with the deterministic model, the same authors have developed the following probabilistic model (Cozens-Roberts et al. 1990):

8 $$ {P}_C\left(t+\Delta  t\right)={P}_C(t)+{k}_f^0{N}_L\left[{R}_T-\left(C-1\right)\right]{P}_{C-1}(t)\Delta  t-\left[{k}_f^0{N}_L\left({R}_T-C\right){P}_C(t)\Delta  t+{k}_r^0C{P}_C(t)\Delta  t\right]+{k}_r^0\left(C+1\right){P}_{C+1}(t)\Delta  t+0\left[{\left(\Delta  t\right)}^2\right] $$

where *P*_*C*_(*t*) represents the probability that *C* receptor–ligand bonds or complexes at time *t* exist, ∆*t* is a small step in time, and *P*_*C*_(*t* + ∆*t*) represents the probability that C complexes exist at time *t* + ∆*t*.

Shear forces exerted on the cell directly affect the residence time of cell’s adhesion to the vessel wall and influences the translational and rotational motion of the CTC inside the vessel (Wirtz et al. 2011).

From a biophysical point of view, Bell’s model has been used to provide a method of investigating the interaction and adhesion of CTCs to the endothelium when fluid flow co-exists (Bell 1979; Bell et al. 1984). Thus, the rates of bond establishment and rupture can be calculated, using the following formula:

9 $$ {k}_{r=}{k}_r^0\mathit{\exp}\left(\frac{r_0F}{k_bT}\right) $$

where *k*_*r*_ is the rate of dissociation, ${k}_r^0$ is the unstressed off-rate, *r*_0_ is the reactive compliance, *F* is the force exerted on the bond, *k*_*b*_ is the Boltzmann constant, and *T* is the temperature.

Data included in the model are external fluid force on one hand and the strength of adhesion receptors that bind to the endothelial cells on the other. When ligand–receptor bonds are subjected to the external forces, such as fluid flow, they should form a stronger adhesion bond than the shear stress in order to remain stable Marshall et al. (2003). Thus, CTCs are involved in a conflict between their adhesion strength and the force exerted by blood flow exerts, with two possible results. If the CTC ligand–receptor adhesion force is weaker than the shear stress, the CTC cannot maintain adhesion. On the contrary, if the CTC adhesion force exceeds shear stress, the cells can efficiently arrest and adhere (Follain et al. 2018; Osmani et al. 2019). Therefore, it is evident that cell motion, which is influenced by the motion of fluid, governs cell and bond position. Stoke’s equation is used to determine fluid's motion.

It has been observed that high shear stress can increase the early arrest of tumor cells by increasing the adhesive points with the EW. Thamilselvan and colleagues, in an in vitro study, suggested that shear is able to reinforce early adhesion between the cell and the EW, through stimulation of a signaling pathway. This involves activation of Src and subsequent reorganization of the actin cytoskeleton and creation of more focal adhesions (Thamilselvan et al. 2007). In the same way, Haier and Nicolson demonstrated the enhancement of cell to EW attachment via FAK phosphorylation in colon carcinoma cells, due to shear stress effect (Haier and Nicolson 2001). This is supported by in vivo data suggesting that mediators inhibiting FAK phosphorylation, significantly diminished the ability of cancerous cells to attach to vasculature within the hepatic microcirculation (von Sengbusch et al. 2005). At the same time, however, high shear stress causes a decrease in the residence time of receptor–ligand pairs and consequently obstructs the formation of stable tumor cell–endothelial cell adhesions (Wirtz et al. 2011). Therefore, in the presence of high shear forces, stronger adhesion is required between the malignant cells and the endothelium in order to get arrested and extravasate. As expected, when experimentally increased flow rates with isobutylmethylxanthine (IBMX), the arrest of CTCs was radically disturbed over a period of 5 min post-injection (mpi) and their mean arrest time reduced. On the contrary, when decreasing flow rates exist, the mean arrest of CTCs significantly increased over a period of 5 mpi (Follain et al. 2018). These results show that, while reduced flow forces lead to a greater arrest probability of CTCs, increased flow forces are capable of disrupting their early arrest (Follain et al. 2018). Hence, it has to be assumed that the probability of a tumor cell’s adhesion to a vessel wall is greater during intermediate values of shear stress and mostly in the arteriovenous junction (AVJ) where a flow drop happens and so normal flow profiles exist (Wirtz et al. 2011; Follain et al. 2018). In addition, upon experimentally intravascular injection of breast cancer cells in zebrafish and mouse brain metastatic models, an intermediate shear stress of 5–7 dyn cm^−2^caused CTC arrest and, consequently, colonization (Follain et al. 2018).

#### Resistance mechanisms to shear forces

Observations in mouse brain, lungs, and rat livers have demonstrated that shear stress tends to increase during intravascular arrest (Gassmann et al. 2009; Kienast et al. 2010; Headley et al. 2016). As a consequence, arrested CTCs experience higher shear forces (Fan et al. 2016), so it is essential that they develop adaptive mechanisms in order to withstand such forces and finally extravasate (Stoletov et al. 2010a, b; Follain et al. 2018; Osmani et al. 2019). It has been demonstrated, using microfluidic approaches, that tumor cells tend to be more resistant to shear stress than normal cells by activating various genetic pathways that can alter cytoskeleton organization, nuclear morphology and adhesive potential (Denais et al. 2016; Raab et al. 2016; Infante et al. 2018). These alterations result in an enhanced cell structure, cell shape deformation, and attachment to the vascular wall (Strilic and Offermanns 2017). These changes also indicate a phenotypic switch from cell–cell adhesion to cell–EW adhesion (Davies et al. 2005).

Initially, while cancer cells become more invasive, they display softer mechanical characteristics that result in larger cell deformations and more pronounced shape changes. There is also evidence of changes in the structural components of the nuclear envelope in various kinds of cancer cells (Chow et al. 2012) that may result in altered mechanical properties. Numerous in vivo studies (Yamauchi et al. 2005; Stoletov et al. 2007; 2010a, b) have showed that metastatic tumor cells are quite deformable, and both the cell cytoplasm and cell nucleus can undergo strong compression and shape deformation in small capillaries. Cancer cells appear as viscoelastic spherical structures, flowing into the vessels that are like fluid-filled tubes. This movement is affected and driven by blood pressure. When entering large blood vessels, cancer cells have a spherical shape (Weiss 1992). Circulating in blood, tumor cells act somehow like leukocytes. These nucleated cells have a diameter of 7–10 μm (Schmid-Schnbein et al. 1980; Ting-Beall et al. 1993) and need to deform to enter and flow into the smallest blood vessels and capillaries (5–9 μm). In the same way, cancer cells display a deformation necessary for their entry into capillaries. Taking into consideration that cancer cells are larger in diameter than small capillaries, they are required to change shape from spherical to cylindrical in order to enter into them. These shape transitions occur while keeping the cell volume constant. Both the cytoskeleton and nucleus play a significant role in the mechanical properties and responses of the cancer cell and the final deformation. Being nucleated cells, tumor cells have a rich network of actin filaments and microtubules connecting the plasma membrane to the nucleus that mainly affect cell’s stiffness (Khismatullin 2009). Cortical tension is created from the attachment of actin filaments to the cell membrane. Every remodeling of actin bundles results in modified structure of actin network, leading to altered stimuli from network to the nucleus and as a consequence to modified cell’s responses (Kaverina et al. 1998; Esue et al. 2006)

**Fig. 6:**
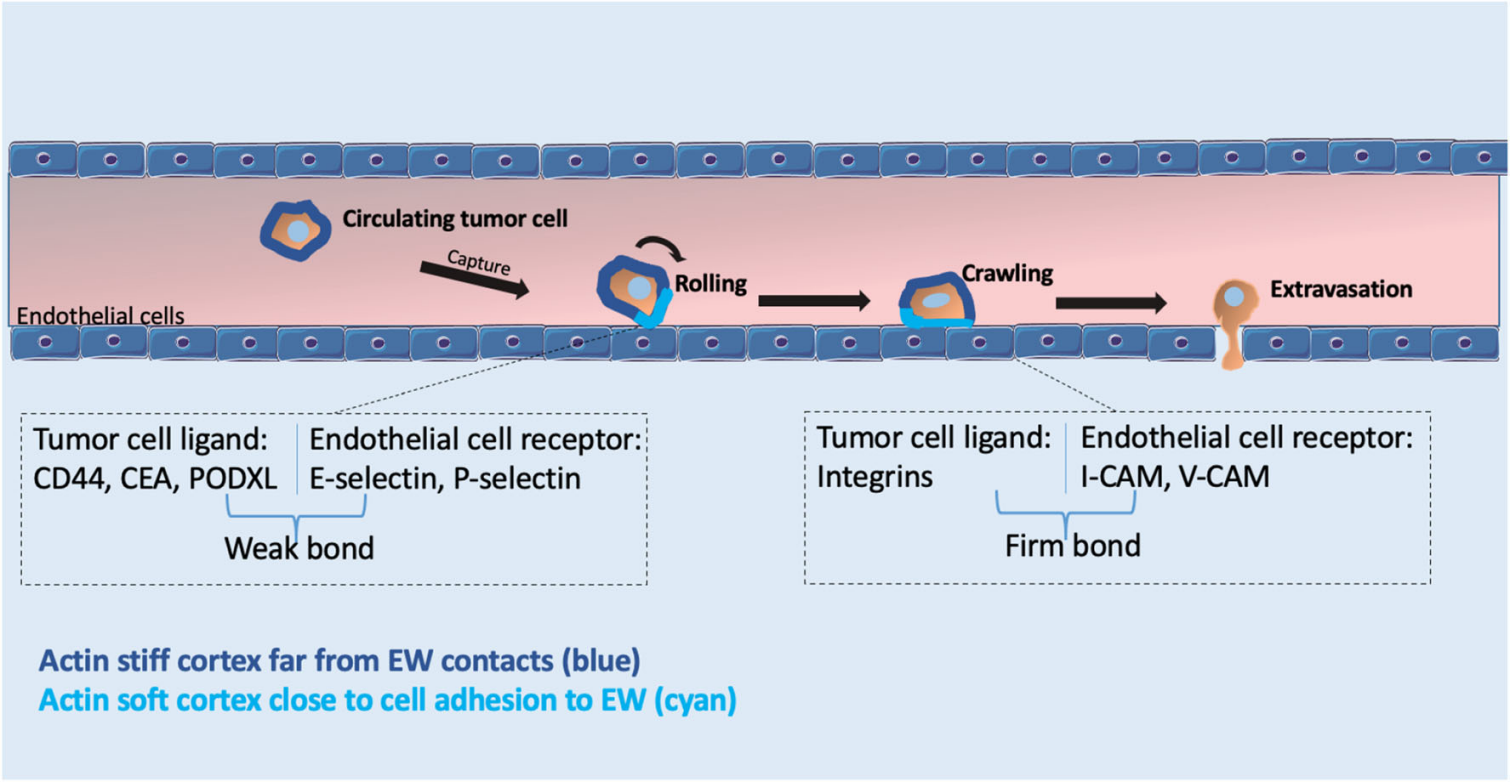
Alterations in cell stiffness and ligand-receptor interactions during transition from floating to crawling. Upon successful intravasation, adhesion of CTCs to the endothelium is important for the cell to withstand shear stress, survive, and extravasate to the secondary tissue. By the time a tumor cell contacts a vessel wall, either a transient or a persistent (firm) adhesion may occur. The ability to arrest depends on the ligand–receptor interactions and binding potential. A weak bond consists of ligands such as CD44, carcinoembryonic antigen (CEA) or podocalyxin (PODXL), and usually selectin receptors. Firm bonds occur between integrins and intercellular adhesion molecule 1 (ICAM1) or vascular cell adhesion molecule 1 (VCAM1) as receptors. During transition from floating to crawling different cytoskeletal properties and cortex stiffness of the CTC appear. A color-coded cell stiffness is presented in figure, where stiff cortex is marked with blue while more deformable cortex as cyan

During this process, as discussed above, the cell circulating with the blood flow needs to switch between various locomotion strategies, from floating to reaching the EW and adhering to the endothelium. Then, the tumor cell has to undergo transitions from rolling to arresting and crawling before it reaches the point where it can finally anchor to the endothelium and transmigrate through the endothelial layer (Nourshargh et al. 2010; Wirtz et al. 2011). In general, previous studies that tried to simulate the process described above, between floating and crawling, found that the circulating tumor cell’s cytoskeleton seems to undergo various modifications concerning its stiffness in different cell compartments (Fuhrmann et al. 2011; Swaminathan et al. 2011; Ketene et al. 2012; Amos and Choi 2021) (Fig. 6). Indeed, it was proved that the cortex actin fibers are subjected to alterations caused both by the blood fluid stress and adhesive contacts with the endothelium (Rejniak 2007; 2012). The model most used to describe this process was the IBCell model of simulation (Rejniak 2007; 2012). While the cell floats, a stiff cytoskeletal cortex is beneficial, or else a very flexible and deformable cell may result in elongation and even cytoplasmic fragmentation, i.e., clasmatosis (Rejniak 2012). Thus, the structure and stiffness of actin plays a significant role in cell survival, during the critical phase of travelling within the blood vessels. It should be noted that a combination of both a soft cortex and soft nucleus may lead to cell damage by the forces exerted within the bloodstream. During initial contact with the endothelial wall and the beginning of the rolling phase, passive tumor cells start to deform creating a large area of contact with the EW. Signaling leads to conformational change of b_2_ integrins in the cell-substrate contact region to a high affinity state (Berger et al. 2002; Lum et al. 2002; Ginsberg et al. 2005; Luo et al. 2007; Shamri et al. 2005) and to cytoskeleton remodeling with an increase in cytoplasmic stiffness (Mofrad and Kamm 2006; Khismatullin 2009). While the cell is in the rolling phase, a fairly stiff actin cortex is preferable, so that the cell can be stressed by the bloodstream without being subjected to extensive deformation. Having both a deformable nucleus and cortex the adhesive connections of the cell to the EW would be easily broken. Such a deformable cell would have greater probability to detach from the EW. By the time the cell is converted from rolling to a cell with anchorage to the vessel wall, the cortex fibers become softened and more flexible along the contact surface with the EW. Therefore, a more deformable cell will have more stable rolling that will help with anchoring. A very weak cytoskeleton would be ineffective for anchoring and crawling, so the cell might become elongated too much and finally carried away by the bloodstream. On the other hand, a very stiff cytoskeleton would not allow the transition from rolling to the next phase. One of the primary reasons causing detachment of a high-stiffness cell from the EW is the rate of bond formation to be less than the rate of bond rupture. In this way the number of unstressed bonds and the contact area will decrease with time until the detachment of the cell. For cell crawling, the actual requirement is the weakening of the binding points with the EW and the establishment of a more flexible and deformable cell structure. This elongation phase, directed by the fluid flow stress, will lead to the formation of a large, flat contact area. Finally, it has been shown that for a circulating tumor cell to exit the circulatory system and migrate to a distant site, it must adhere to the lumen of the vessel wall and squeeze through the vasculature to seed within a secondary tissue (Janmey et al. 2016).

#### Fluid flow and CTC extravasation

Upon survival and stable attachment of CTCs to the vessel wall, the next target of tumor cells is to extravasate to secondary tissues. Shear forces also play a significant role in this stage of metastasis. As already discussed, intermediate flow forces allow the arrest of CTCs to the EW. Additionally, intermediate forces promote adhesive points between CTCs and the EW through stimulation of signaling pathways (Wirtz et al. 2011; Follain et al. 2018). Data suggest that although early arrest and adhesion occurs in reduced and intermediate flow regions respectively, sufficiently intermediate and high flow regions are the most favorable for successful extravasation (Lapis et al. 1988; Follain et al. 2018). It has been documented that increased flow stimulates endothelial remodeling, which is an essential prerequisite for metastasis formation (Lapis et al. 1988; Follain et al. 2018). Therefore, published data suggest that increased fluid flow promote the extravasation of CTCs.

#### Role of cell–platelet interactions in the blood

**Fig. 7:**
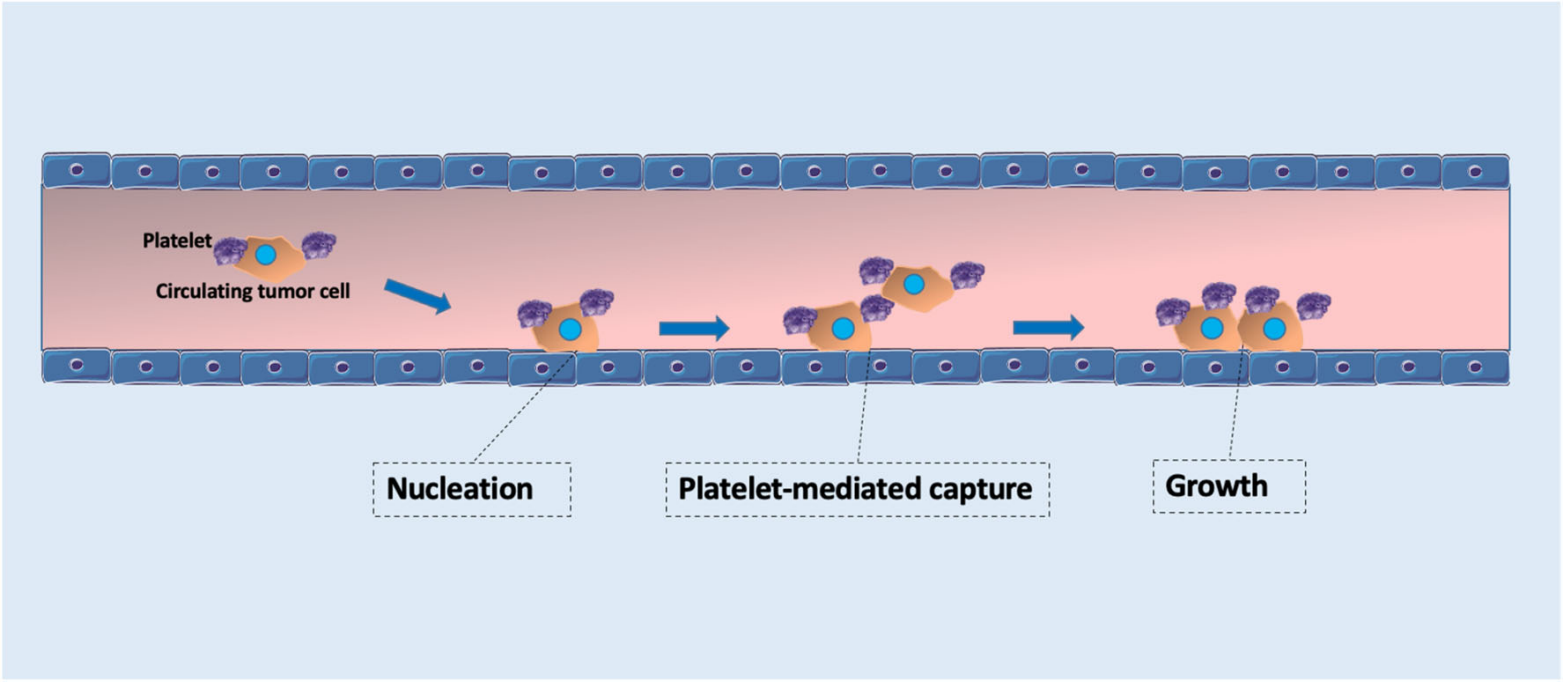
Platelet-mediated tumor growth. During their circulation, tumor cells (CTCs) develop protective mechanisms against shear forces. CTCs bind to platelets, forming tumor cell–platelet microaggregates that enhance arrest through platelet-mediated capture. A tumor cell–platelet microaggregate adheres to the endothelium and serves as a “nucleus” to capture flowing cells. Τhese cells subsequently attach to the EW next to the already attached microaggregate through a platelet-bridging mechanism. This process is known as “nucleation”

CTCs are not as capable as blood cells at withstanding shear forces (Moazzam et al. 1997; Guido and Tomaiuolo 2009; Xiao et al. 2017). Interestingly, CTCs have developed mechanisms of interaction with blood components which protect them against shear forces and mechanical stress-induced cell death. For example, CTCs can escape immune control by binding to platelets, forming tumor cell-platelet microaggregates. Studies have shown that when platelets are depleted, either due to pharmacological or genetic issues, the metastatic process becomes difficult (Gasic et al. 1968; Camerer et al. 2004). On the other hand, when there is adequate platelet supply, metastasis returns to normal, according to studies based on a mouse model (Karpatkin et al. 1988). Therefore, it is believed that the formation of adhesive clusters of CTCs with platelets also provides protection of CTCs from an immune attack (Nieswandt et al. 1999; Palumbo et al. 2005). In addition, clusters with platelets also play a role in the adhesion of tumor cells to the vessel wall by releasing a number of bioactive agents, such as vascular endothelial growth factor (VEGF) in the endothelium. This causes an increase in vascular permeability, which assists the extravasation of cancer cells (Felding-Habermann et al. 1996; Burdick and Konstantopoulos 2004; Gay and Felding-Habermann 2011). The way that tumor cell–platelet complexes may enhance arrest is called platelet-mediated capture, a process analogous to nucleation and growth. The growth process starts with the formation of a small cluster consisted of platelets linked to a cancer cell that is already adhered to the endothelium. This cluster serves as a “nucleus” to capture free-flowing cells that subsequently attach to the blood vessel wall directly next to the already adherent cell of the cluster. This “nucleation” mechanism results in the formation of growing clusters of cancer cells adherent to the EW (Fig. 7). From a molecular aspect this process is primarily dependent on P-selectin glycoprotein ligand 1 (PSGL1) or platelet endothelial adhesion molecule 1 (PECAM1) (Frenette et al. 1995; Reymond et al. 2013). Although the influence of platelets to the metastatic potential of CTCs has been demonstrated, additional research is needed to clarify how platelets behave when adhered to CTCs under shear stress, and the downstream effect on CTC survival.

## Conclusion

This review has highlighted some of the biophysical interactions between components of the tumor microenvironment, as well as the cell’s mechanical alterations associated with cancer progression. One of the most interesting points is the understanding that tumor growth, invasion and metastasis are directly linked to the ability of the cell’s components to sense and adapt to mechanical stimuli from their environment. Metastasis is a “forced journey” consisting of changes in tumor cell shape, intracellular mechanical properties and motility. These alterations are mainly regulated by the cytoskeleton. All of these changes are combined with the molecular mechanisms regulating cellular responses associated with cancer.

This review has also examined biological fluids and their mechanical effect to the survival and metastatic potential of CTCs. Yet, the process of tumor cells circulating in the bloodstream, their physical ability to survive during this process and become able to successfully extravasate to secondary tissues is still under investigation. However, fluid force is a factor which actively influences both stable arrest and extravasation of CTCs, preceding metastatic outgrowth and so is a key component affecting tumor aggression and progression. An in-depth understanding of the metastatic mechanisms is essential in developing successful therapeutic treatments. More work needs to be done to understand how and where to best intervene in order to combat metastatic spread of disease and perhaps turn cancer into a chronic but manageable disease.
